# Mucosal vaccine delivery by non-recombinant spores of *Bacillus subtilis*

**DOI:** 10.1186/s12934-014-0115-2

**Published:** 2014-08-12

**Authors:** Ezio Ricca, Loredana Baccigalupi, Giuseppina Cangiano, Maurilio De Felice, Rachele Isticato

**Affiliations:** Department of Biology, Federico II University of Naples, via Cinthia, 80126 Naples, Italy

**Keywords:** Vaccine vehicles, Vaccine delivery, Drug delivery, Surface display

## Abstract

Development of mucosal vaccines strongly relies on an efficient delivery system and, over the years, a variety of approaches based on phages, bacteria or synthetic nanoparticles have been proposed to display and deliver antigens. The spore of *Bacillus subtilis* displaying heterologous antigens has also been considered as a mucosal vaccine vehicle, and shown able to conjugate some advantages of live microrganisms with some of synthetic nanoparticles. Here we review the use of non-recombinant spores of *B. subtilis* as a delivery system for mucosal immunizations. The non-recombinant display is based on the adsorption of heterologous molecules on the spore surface without the need of genetic manipulations, thus avoiding all concerns about the use and environmental release of genetically modified microorganisms. In addition, adsorbed molecules are stabilized and protected by the interaction with the spore, suggesting that this system could reduce the rapid degradation of the antigen, often observed with other delivery systems and identified as a major drawback of mucosal vaccines.

## Introduction

Currently licensed vaccines are delivered by one of five main administration routes. The intramuscular route is the most common one and several vaccines, such as hepatitis A and B, rabies, influenza, diphtheria, tetanus and pertussis, are delivered by this approach. Subcutaneous and intradermal routes are also common and used, for example, for measles-mumps-rubella-yellow fever and BCG-rabies vaccinations, respectively. The remaining two routes deliver antigens to the nasal or intestinal mucosal surfaces. The intranasal route is used, for example, for live attenuated influenza viruses while the oral route is used for poliomyelitis, cholera, rotavirus and typhoid fever vaccinations.

Mucosally administered vaccines have a number of potential advantages over injectable vaccines: no risk of transmission of blood-borne diseases; no need of trained personnel to administer and the possibility to elicit an immune response at the portal of entry of most pathogens [1]. However, despite these potential advantages and early success with the oral polio vaccine more than 50 years ago, injectable vaccines are widely more common than mucosal ones [2]. This is in part due to a number of drawbacks associated with currently available mucosal vaccines, such as poor antigen adsorption, rapid antigen degradation on the mucosal surfaces and lack of safe and effective mucosal adjuvants [2]. Recent advances in mucosal immunology, and recent success with the development of mucosal vaccines against influenza and rotavirus infections have renewed interest in the development of a new generation of mucosal vaccines. Public health organizations are urging the scientific community to focus on the development of efficient systems to deliver antigens to mucosal sites that facilitate uptake by local antigen-presenting cells, and on the discovery of safe and effective mucosal adjuvants that enhance the protective mucosal immune response.

We review here the use of non-recombinant spores of *Bacillus subtilis* as a delivery system for mucosal immunizations. Spores of *B. subtilis* are extremely resistant and stable, easy to manipulate and are known to interact with immune cells inducing protective, antigen-specific immune responses [3]. A variety of antigens have been displayed on the surface of recombinant *B. subtilis* spores and used to immunize animal models through the nasal and/or oral route [4]. Like other display systems, also the spore-based approach rely on the genetic engineering of the host. This raises concerns over the use of live genetically modified microorganisms, their release into nature and their clearance from the host following delivery [5]. To overcome this obstacle a non-recombinant approach to display heterologous proteins on the spore surface has been recently proposed.

### The spore of *Bacillus subtilis*

Spores are quiescent cells mainly produced by members of two genera of Gram-positive bacteria, the aerobic or facultative anaerobic *Bacilli* and the anaerobic *Clostridia* [6]. The common feature of these organisms is the ability to respond to harsh environmental conditions differentiating a spore from a vegetative cell. Because of its peculiar structure the spore is extremely stable and survives indefinitely to the absence of water and nutrients, to the presence of lytic enzymes and toxic chemicals, of UV irradiation and to extremes of temperature and pH [7]. However, in the presence of water, nutrients and favourable environmental conditions, the spore germinates generating a cell able to duplicate vegetatively and, eventually, to re-sporulate. The processes of sporulation and germination in *Bacillus subtilis*, the model system for spore-formers, have been recently reviewed [8,9].

In *B. subtilis* the spore structure has been studied in details and shown to be formed by a dehydrated cytoplasm (core) surrounded by several protective layers: the thick peptidoglycan-like cortex, the multilayered, proteinaceous coat and the crust, the outermost layer formed of proteins and glycans [7]. Interestingly, several spore surface proteins have the ability to self-assemble [10,11] and the entire spore can self-assemble into functional structures [12].

Despite their metabolic quiescence, spores are dynamic structures able to respond to changes in relative humidity by expanding and shrinking [13]. It has been measured that in response to humidity the *B. subtilis* spore can change its diameter by as much as 12% [14,15]. Such variations have been recently used for energy conversion and have proposed the spore as a building block for novel stimuli-responsive materials with potential applications in energy harvesting and storage [12].

Spores of several aerobic species are ubiquitous in nature [16]. In recent years large numbers of aerobic spore-formers, including members of the *B. subtilis* species, have also been found associated to the human and animal gut [17,18]. It has been shown that ingested spores of *B. subtilis* safely transit the stomach, germinate and proliferate in the upper part of the intestine [19]. In the lower part of the intestine the cells sporulate again, thus performing an entire life cycle in the animal gastro-intestinal tract (GIT) [20]. In the GIT *B. subtilis* interacts with intestinal epithelial and immune cells [21–23], contributes to the normal development of the gut-associated lymphoid tissue (GALT) [24] and protects the host from enteropathogens [25]. Such interactions with intestinal cells are the base for the use of several isolates of *B. subtilis* in commercial probiotic preparations (Table 1) [20].

**Table 1:** **Examples of commercial products containing spores of** ***B. subtilis*** **for human or animal use** ^**1**^

**Product**	**Spores/dose**	**Manufacturer**	**Use**
Bibactyl	10^7^-10^8^	Tediphar Corporation (VietNam)	Human
Bio-Kult	NS^2^	Protexin Health Care (UK)	Human
Biobaby	3 × 10^6^ plus other bacteria	Ildong Pharma (Korea)	Human
BioGrow	1.6 × 10^9^ plus other bacteria	Provita Eurotech (UK)	Poultry, calves, swine
BioPlus	1.6 × 10^9^ plus other bacteria	Christian Hansen (Denmark)	Piglets, poultry
Biosporin	NS^2^	Bioparm (Ukrine)	Human
Biostart	NS^2^	Microbial Solutions (South Africa)	Aquaculture
Biosubtyl DL	10^7^-10^8^	IVAC (VietNam)	Human
BioZyme-Aqua	1 × 10^8^	Sino-Aqua Corp. (Taiwan)	Aquaculture
Ildong Biovita	3 × 10^6^ plus other bacteria	Ildong Pharma (Korea)	Human
Lactipan Plus	2 × 10^9^	Ist. Biochimico Italiano (Italy)	Human
Medilac-Vita	1 × 10^8^ plus other bacteria	Hanmi Pharmaceutical (China)	Human
Nature’s First Food	NS^2^	Nature’s First Law (USA)	Human
Neolactoflorene	NS^2^	Newpharma (Italy)	Human
Pastylbio	1 × 10^8^	Pasteur Institute (VietNam)	Human
Primal defense	NS^2^	Garden of Life (USA)	Human
Promarine	NS^2^	Sino-Aqua (Taiwan)	Aquaculture

^1^Adapted from [20]; ^2^Not Specified.

### Recombinant spore-surface display

**Figure 1:**
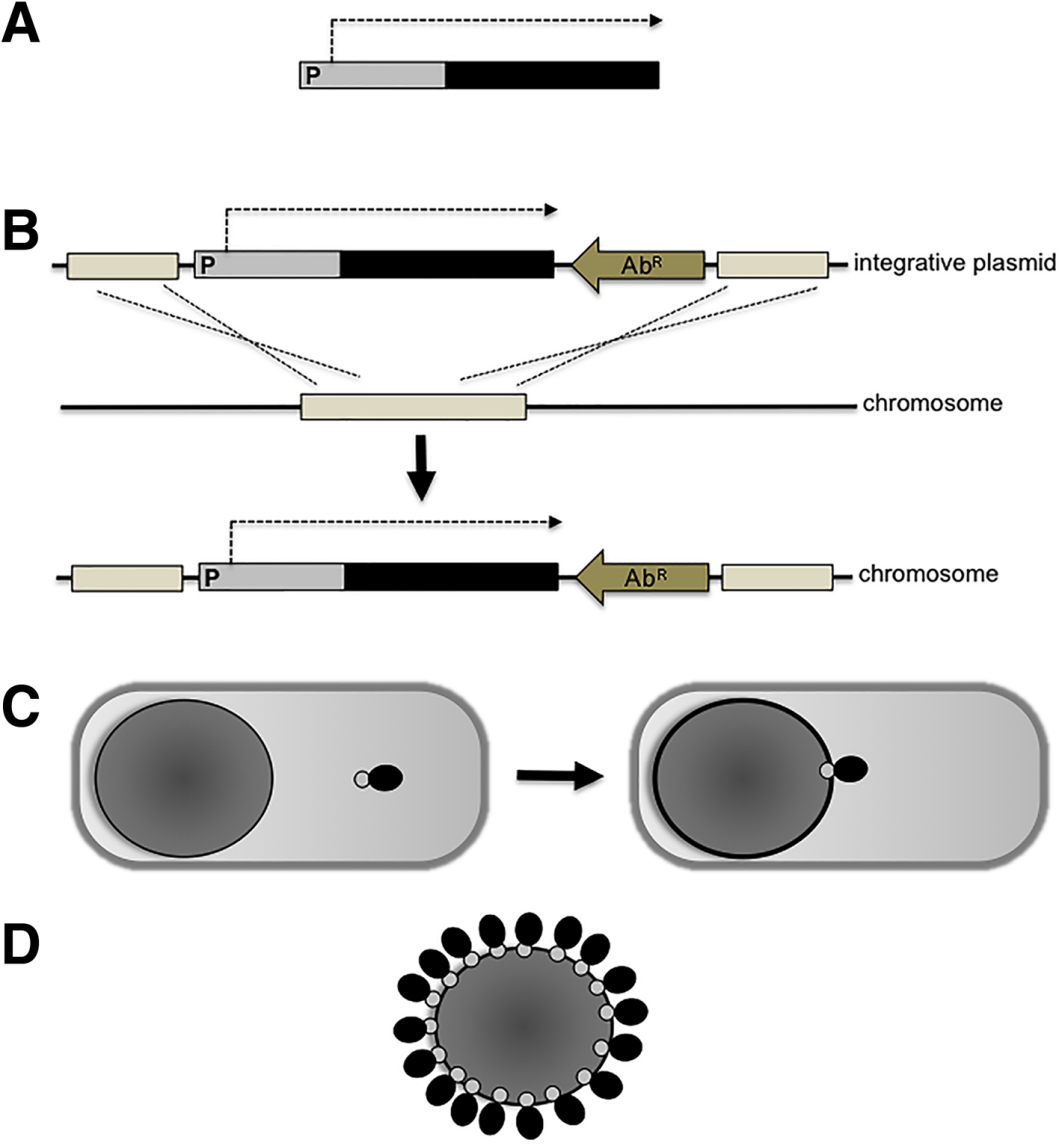
**Strategy of recombinant spore-surface display. A)** A gene fusion is constructed between DNA coding for a spore surface protein (gray) and for an antigen (black). The fusion is under the transcriptional and translational signals of the spore surface gene. **B)** The gene fusion is cloned into an integrative plasmid next to an antibiotic-resistance gene cassette (Ab^R^) and between two parts of a non-essential gene of *B. subtilis*. The gene fusion is integrated on the *B. subtilis* chromosome by a double cross over between homologous DNA present on the integrative plasmid and on the chromosome, interrupting the non-essential gene. **C)** During sporulation the gene fusion is expressed in the mother cell and the chimera assembled around the forming spore. **D)** At the end of sporulation the mother cell lyses releasing the mature spore with the antigen stably anchored to its surface.

The rigidity and compactness of the spore surface together with its proteinaceous composition suggested the possibility of using surface proteins to fuse and anchor heterologous proteins. The genetic system developed to this aim is summarized in Figure 1 and is based on the: i) construction of a gene fusion between DNA fragments coding for an antigen and a surface protein, with transcriptional and translational signals of the latter controlling the expression of the fusion; ii) integration of the fusion on the *B. subtilis* chromosome to grant genetic stability; iii) expression of the gene fusion in the mother cell and the assembly of the chimera around the forming spore; iv) purification of the recombinant spore carrying the chimera stably associated to the spore surface. This system was initially developed by using the spore surface protein CotB as a carrier and the C fragment of the tetanus toxin (TTFC) of *Clostridium tetani* as a model antigen and was shown able to display an average of 1.5 × 10^3^ recombinant molecules per spore [26]. Spore-exposed TTFC molecules were able to induce an antigen-specific immune response and to protect orally immunized mice in challenge experiments [27]. The immune response induced by spores displaying TTFC was not dependent on the ability of the spore to germinate in the GIT of the immunized animals, as shown by immunization experiments with mutant spores displaying TTFC but unable to germinate [28]. Over the years the same approach has been utilized to display several different antigens with various spore surface proteins as carriers (for a recent review see 4). In several cases the recombinant spores have been tested as mucosal vaccines in animal models and proved able to induce specific and protective immune responses (for a review see 3). Recently, a set of plasmids have been developed to facilitate the construction of gene fusions using selected *cot* genes as carriers [29]. A useful development of some of these plasmids is that they allow the integration on the *B. subtilis* chromosome without the need to select for an antibiotic marker. By this system the recombinant strains display a heterologous antigen but do not contain an antibiotic-resistance gene [29].

### Non-recombinant spore-surface display

**Figure 2:**
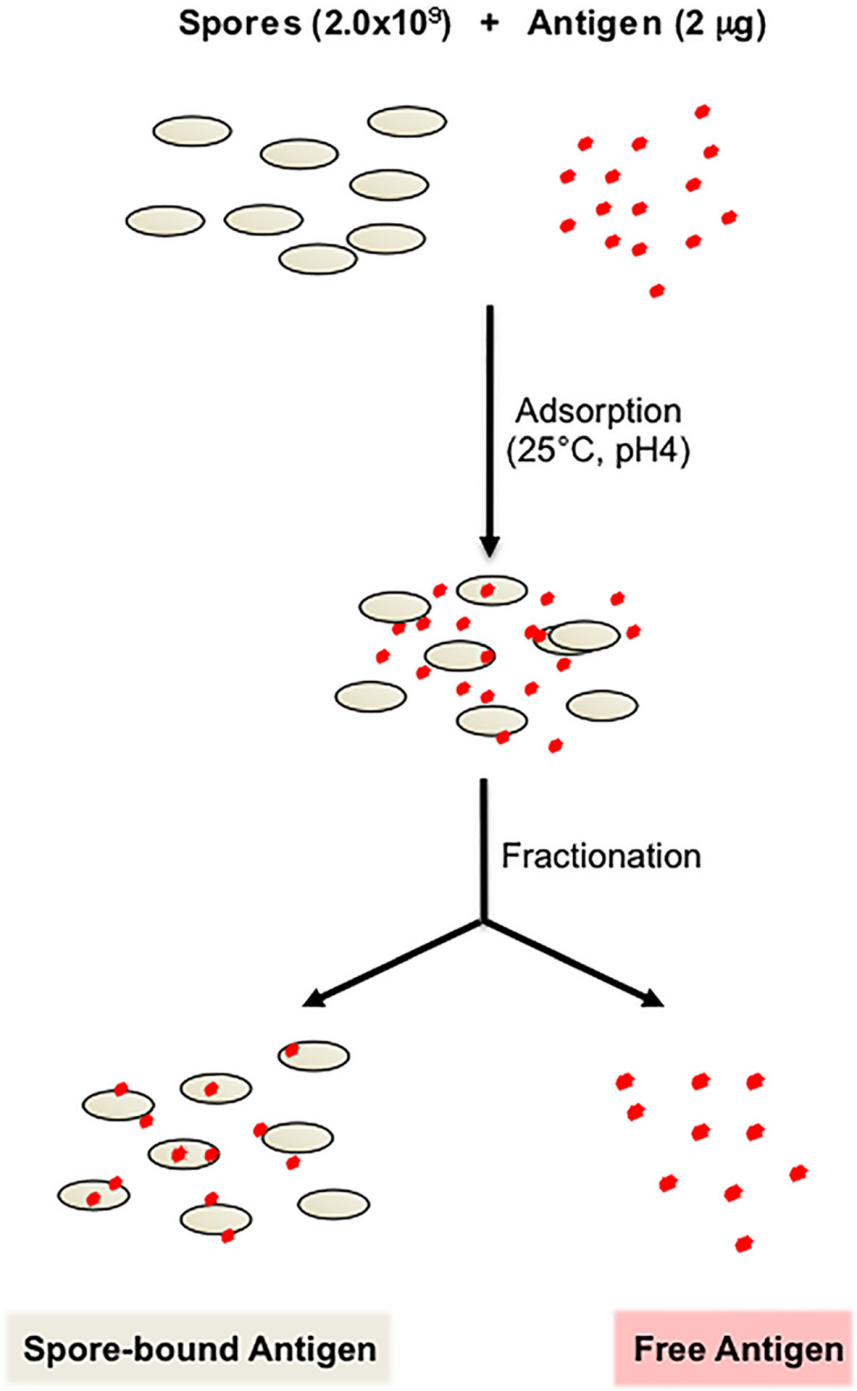
**Strategy of non-recombinant spore-surface display.** Purified spores and antigen are reacted at 25°C in an acidic reaction buffer (pH 4.0). After the adsorption reaction spore-bound and free antigen are separated by centrifugation.

The display strategy summarized in Figure 1, even when it does not involve an antibiotic-resistance gene [29], relies on the genetic engineering of the host and involves the release into nature of a recombinant microorganism. This is considered as a major drawback, especially when the display system is designed for human or animal use [5]. Therefore, non-recombinant approaches are highly desirable and their development is strongly encouraged by control agencies [5]. In this context, a non-recombinant approach to display heterologous proteins on the spore surface has also been developed. As summarized in Figure 2, upon incubation of spores with a purified antigen part of the antigen molecules are stably bound to the spore surface. Huang et al. [30] have reported that four antigens (TTFC of *C. tetani*, PA of *B. anthracis*, Cpa of *C. perfringens* and glutathione S transferase from *Shistosomas japonica*) were efficiently adsorbed to spores. Adsorption was more efficient at acidic conditions (pH 4.0) and less efficient or totally inhibited at pH 7.0 or 10.0, respectively. Adsorption was not dependent on any specific coat proteins but, rather, due to a combination of electrostatic and hydrophobic interactions between spores and antigen [30]. Importantly, antigen-adsorbed spores were able to induce specific and protective immune responses in nasally immunized mice [30].

In a similar way also a model enzyme, the β-galactosidase of *Alicyclobacillus acidocaldaricus,* was efficiently adsorbed to *B. subtilis* spores [31]. Also in this case adsorption was dependent of the pH of the binding solution and was more efficient at acidic than neutral or basic conditions. This study, although not performed with an antigen, revealed some properties of spore adsorption that could turn out potentially useful for the development of new mucosal vaccines: 1) the adsorbed enzyme was more stable than the unbound, free enzyme at both high temperatures and low pH values, suggesting that the interaction with the spore stabilizes and protects the heterologous protein; 2) mutant spores with a strongly altered surface adsorbed the enzyme more efficiently than isogenic wild type spores [31]. *B. subtilis* spores are known to be negatively charged and in an aqueous environment behave like almost infinite ionic reservoirs accumulating billions of protons (approximately 2 × 10^10^ per spore) [32]. An expected consequence of the reduced number of free protons in solution is a pH increase and, indeed, 2 × 10^9^ spores were able to raise the pH of pure water from 5.98 to over 7.00 [31]. The same amounts of mutant spores with either a strongly altered (*cotH*) or a totally lacking (*cotE*) outer coat did not alter the pH of pure water, indicating that they did not attract protons and, therefore, are not (or less) negatively charged [31]. Since these mutant spores adsorb β-Gal more efficiently than wild type spores, the negative electric charge of the spore is not a major determinant of β-Gal adsorption [31]. More recently another enzyme, the cellobiose 2-epimerase of *Caldicellulosiruptor saccharolyticus* has been adsorbed on the spore surface [33]. Also in this case, the spore-immobilized enzyme showed a higher pH and thermal stability than the free enzyme. Adsorption was very stable and the enzyme detached from the spore surface only by a drastic treatment with 1.0 M NaCl at pH 1.0 [33].

Live as well as heat-inactivated spores were also shown able to bind influenza H5N1 virions (NIBRG-14; clade 1) [34]. Groups of mice intranasally immunized with killed spores adsorbed with NIBRG-14 were able to fully protect the animals in a challenge experiment with a lethal dose (20 times the LD50) of the virus. Particularly interesting is the observation that killed spores without any adsorbed virion were able to partially protect (60%) the animals through the induction of an innate immune response, suggesting for the spore an adjuvant role for H5N1 vaccination [34].

Spores of *B. subtilis* have also been used to adsorb two *Mycobacterium tuberculosis* antigens: i) MPT64, a 25-kDa secreted protein, not produced by attenuated strains, characterized by a highly immunogenic activity and shown able to confer partial protection in challenge experiments with a mouse models [35]; and ii) the chimera Acr-Ag85B, formed by two antigens, Acr preferentially recognized by latently infected individuals and Ag85B, one of the most protective antigens of *M. tuberculosis.* As in the previous cases, adsorption of the two *M. tuberculosis* antigens was pH-dependent. Intranasally administered spores were able to reach the alveoli and to induce both humoral and cellular immune responses [36]. Immunized animals were protected in a challenge experiment and presented reduced mycobacterial loads in their lungs and spleens, confirming that mucosal vaccinations are particularly effective against pathogens entering the animal body through the mucosal surfaces [36].

### Recombinant vs. non-recombinant spore-surface display

A recent report has compared the efficiency of recombinant and non-recombinant spore display using as a model antigen the binding subunit of the heat-labile toxin (LTB) of *Escherichia coli* [37]. LTB displayed by both strategies was able to induce a specific immune response in mucosally immunized mice [37,38]. An average of 9.6 × 10^-5^ pg of LTB/spore, corresponding to approx. 200 ng of LTB in a dose of 2 × 10^9^ spores, were displayed by the recombinant approach (CotC-LTB), while the same number of spores was able to display up to 5.5 μg of LTB by the non-recombinant approach [37]. The 25-fold higher efficiency of display obtained with the non-recombinant strategy than with the recombinant one was further improved using mutant spores with an altered outer coat (*cotH* mutant spores). 2 × 10^9^*cotH* spores adsorbed approx. 14 μg of LTB, about 70-fold more efficient than the antigen displayed by the recombinant approach [37]. The increased efficiency of display of non-recombinant vs. recombinant approach is particularly relevant since it allows to reduce the number of spores needed to induce an immune response. Effective immunizations have been obtained in mice dosing the animals with at least nine doses of 1.0 × 10^10^ recombinant spores [38]. Scaling up that number to immunize humans would be extremely difficult or even not realistic, making necessary to use a more efficient display systems.

**Figure 3:**
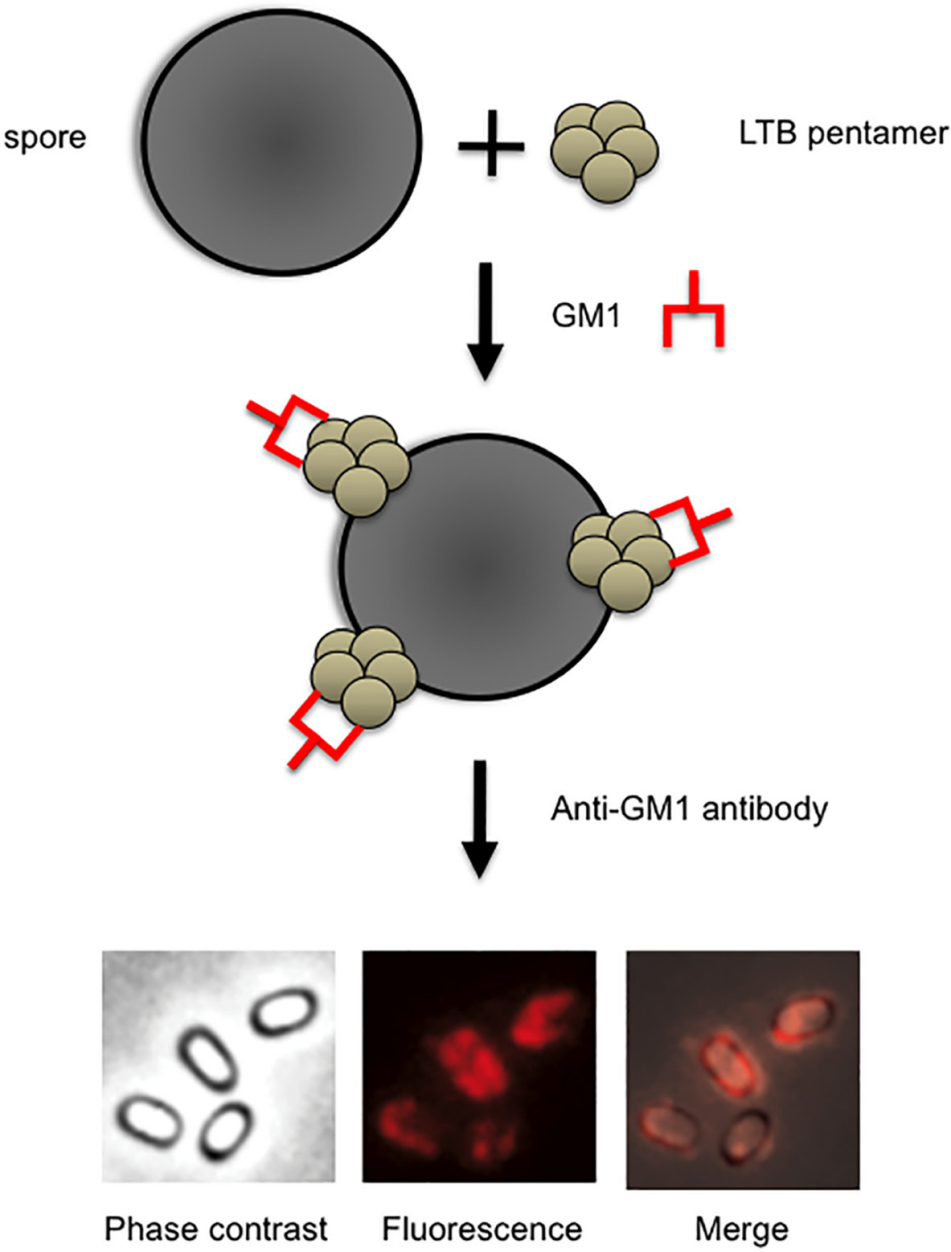
**Display of a multimeric antigen on the spore surface.** Purified spores are reacted with the LTB pentamers. Spore-adsorbed pentamers are reacted with the purified receptor (GM1). Spores are visualized by immunofluorescence microscopy with anti-GM1 primary antibody and Texas red conjugated secondary antibody [36]. The same microscopy field is observed by phase contrast and fluorescence microscopy. The merge of the two images is also shown.

An additional advantage of the non-recombinant system over the recombinant one is that only the former approach allows the display of a multimeric antigen in its native form. LTB, like the B subunit of the cholera toxin, forms pentamers and only as a pentamer can bind its natural receptor, the GM1 ganglioside [39]. While only LTB monomers can be displayed by the recombinant approach on the surface of *Streptococcus gordonii* [40] or of *B. subtilis* spores, LTB pentamers can be displayed by the non-recombinant approach on the spore surface (Figure 3) [37]. This aspect is crucial since the immunostimulatory activity of LTB largely depends on its ability to bind to its receptor, which only recognizes the pentamer via non-covalent associations. Pentamer formation and interaction with GM1 result in enhanced targeting and access to MHC compartments [41] with the consequent increased activation of antigen presenting cells and T cells [42].

### Advantages of the non-recombinant spore-based delivery system

The use of *B. subtilis* spores, both by the recombinant and non-recombinant approach, has several advantages over other cell- or phage-based display systems. A first advantage comes from the safety of *B. subtilis* spores for human use. The widespread utilization of spores of this species in commercial probiotic preparations and in traditional food preparations (Table 1) represents an exceptional safety record for the *B. subtilis* species. Other advantages come from the well documented robustness of the spore which grants high stability to the display system even after a prolonged storage [43]. The stability of the delivery system is an important requirement in developing new mucosal vaccine, especially if intended for vaccination programmes in developing countries, where poor distribution and storage conditions are major limitations.

To these advantages the non-recombinant display approach adds additional favourable properties. No genetic manipulation is required for the display, eliminating concerns over the use and release of recombinant microorganisms. Spore-adsorbed proteins are stabilized and protected by the interaction with the spore and are more stable that free proteins to high temperatures and acidic pH conditions [31], suggesting that spore-exposed antigens may have a longer half-life at mucosal surfaces.

The non-recombinant approach is more efficient than the recombinant spore display system, allowing to use up to 70-fold less spores to deliver a similar amount of antigen. Finally, only the non-recombinant approach allows the disply of multimeric antigens in their native form, in turn ensuring the recognition of the natural receptor and a proper activation of the immune system [37].

### Future perspectives

Future developments of the non-recombinant spore display system will necessarily have to be based on a better understanding of the mechanisms involved in spore adsorption. A combination of electrostatic and hydrophobic interactions between spores and passengers have initially been suggested as responsible of the adhesion [30]. However, in the case of β-galactosidase adsorption the role of electrostatic force has been shown to be not predominant [31]. It is possible that adsorption is somehow multifactorial and not due to a single mechanism, however, whether other factors (e.g., van der Waals and capillary forces) are also involved, how the involved forces are affected by external factors such as humidity, or by properties of the passenger protein are all relevant questions that still need to be addressed. Relevant in this frame is the observation that spores can be studied at a single-cell level by the use of optical tweezers, thus opening to the possibility of a deep characterization of the physico-chemical properties of the spore surface [44].

**Figure 4:**
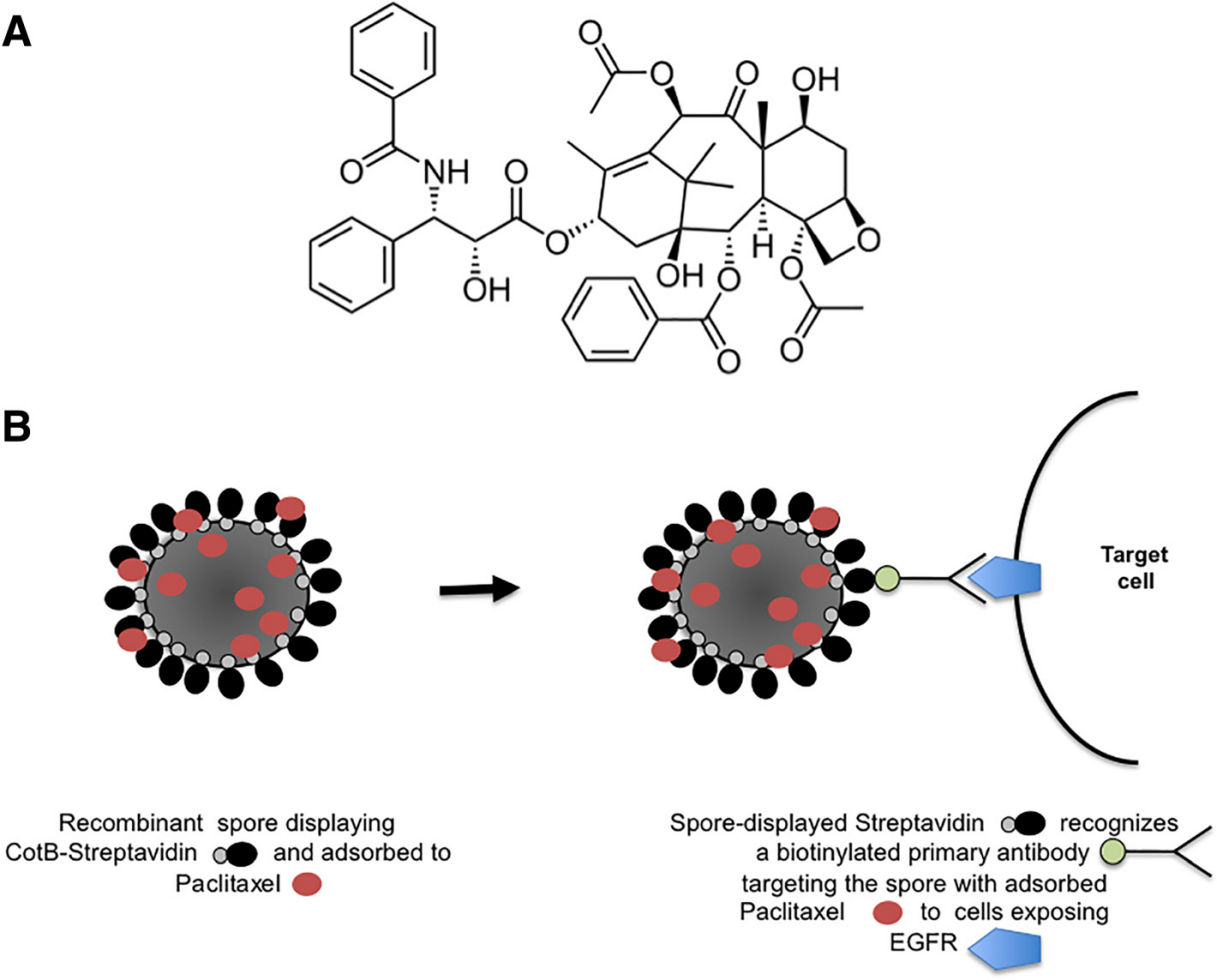
**Combined recombinant and non-recombinant spore-surface display. A)** The diterpen paclitaxel, used as an example of non-proteinaceous molecule adsorbed to the spore. **B)** Spore displaying streptavidin by the recombinant approach and paclitaxel by the non-recombinant one. By streptavidin-biotin interaction the spore binds to a biotinylated anti-EGFR primary antibody targeting the spore to the EGFR-exposing cell [43].

A future and exciting extension of recombinant and non-recombinant spore display systems comes from a recent report showing that: i) the two approaches can be used in combination, and ii) non-proteinaceous molecules can also be adsorbed to the spore surface [45]. The diterpen paclitaxel, a mitotic inhibitor used in cancer therapy (Figure 4A), was adsorbed on *B. subtilis* spores already displaying streptavidin as a chimeric fusion to the spore surface protein CotB [45]. The recombinant spores were able to bind a primary biotinylated antibody specifically reacting with the human epidermal growth factor receptor (EGFR, Cetuximab), thus targeting spores and molecules adsobed to them to the surface of cells exposing EGFR (Figure 4B) [45]. By this approach spores displaying streptavidin can bind any biotinylated antibody, potentially targeting spores and adsorbed molecules to any potential target cell.
